# Endothelial protein kinase MAP4K4 promotes vascular inflammation and atherosclerosis

**DOI:** 10.1038/ncomms9995

**Published:** 2015-12-21

**Authors:** Rachel J. Roth Flach, Athanasia Skoura, Anouch Matevossian, Laura V. Danai, Wei Zheng, Christian Cortes, Samit K. Bhattacharya, Myriam Aouadi, Nana Hagan, Joseph C. Yawe, Pranitha Vangala, Lorena Garcia Menendez, Marcus P. Cooper, Timothy P. Fitzgibbons, Leonard Buckbinder, Michael P. Czech

**Affiliations:** 1 Program in Molecular Medicine, Worcester, Massachusetts 01605, USA; 2 Cardiovascular and Metabolic Research Unit, Cambridge, Massachusetts 02139, USA; 3 Worldwide Medicinal Chemistry Pfizer, Cambridge, Massachusetts 02139, USA; 4 Division of Cardiovascular Medicine, University of Massachusetts Medical School, Worcester, Massachusetts 01605, USA

## Abstract

Signalling pathways that control endothelial cell (EC) permeability, leukocyte adhesion and inflammation are pivotal for atherosclerosis initiation and progression. Here we demonstrate that the Sterile-20-like mitogen-activated protein kinase kinase kinase kinase 4 (MAP4K4), which has been implicated in inflammation, is abundantly expressed in ECs and in atherosclerotic plaques from mice and humans. On the basis of endothelial-specific MAP4K4 gene silencing and gene ablation experiments in *Apoe*^−/−^ mice, we show that MAP4K4 in ECs markedly promotes Western diet-induced aortic macrophage accumulation and atherosclerotic plaque development. Treatment of *Apoe*^−/−^ and *Ldlr*^−*/*−^ mice with a selective small-molecule MAP4K4 inhibitor also markedly reduces atherosclerotic lesion area. MAP4K4 silencing in cultured ECs attenuates cell surface adhesion molecule expression while reducing nuclear localization and activity of NFκB, which is critical for promoting EC activation and atherosclerosis. Taken together, these results reveal that MAP4K4 is a key signalling node that promotes immune cell recruitment in atherosclerosis.

## Abstract (web summary)

Atherosclerosis is an inflammatory disease with limited therapeutic options. Here, the authors show that protein kinase MAP4K4 regulates vascular inflammation underlying atherosclerotic plaque development and that its inhibition prevents the disease and promotes lesion regression in mice, proposing a new atherosclerosis treatment.

## Graphical abstract

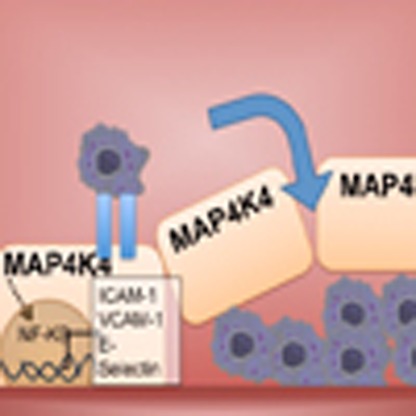

Atherosclerosis is a progressive disease that is often considered an inflammatory response to chronic vessel injury, which is mediated in part by perturbed vascular flow and oxidized lipoprotein-mediated vascular inflammation. In the case of acute injury, endothelial cytokine secretion and leukocyte adhesion molecule upregulation promotes monocyte extravasation from blood vessels into the sub-endothelial space; however, in atherosclerosis, this becomes pathological as macrophages accumulate as foam cells^1^,^2^,^3^. Cell surface adhesion molecule upregulation, particularly vascular cell adhesion molecule-1 (VCAM-1), is required to mediate monocyte extravasation in response to acute injury. However, chronic vascular inflammation can increase VCAM-1 expression in the vessel wall, which has been implicated in atherosclerotic lesion initiation and progression^4^,^5^,^6^,^7^,^8^. This series of dysfunctions contributes to cardiovascular events such as myocardial infarct, ischaemia and stroke, which are leading causes of death^1^,^2^,^3^.

Mitogen-activated protein kinase kinase kinase kinase 4 (MAP4K4; also known as Nck-interacting kinase (NIK) and hepatocyte progenitor kinase-like/germinal centre kinase-like kinase (HGK)) belongs to the sterile-20 protein kinase family and is most closely related to protein kinases Misshapen-like kinase 1 (MINK) and Traf2 and Nik-interacting kinase (TNIK). Though original reports demonstrated a role for MAP4K4 upstream of the c-Jun NH_2_-terminal kinase (JNK) signalling cascade as a MAP4K^9^,^10^,^11^, studies from our laboratory have demonstrated that MAP4K4 is not in fact required for MAPK activation^12^,^13^,^14^. In endothelial cells (ECs), tumour necrosis factor (TNF)-α and other cytokines induce the expression of several inflammatory genes including those encoding leukocyte adhesion molecules and chemokines^15^. Furthermore, inhibiting upstream signalling cascades that promote adhesion molecule expression such as nuclear factor kappa light-chain enhancer of activated B cells (NFκB) specifically in ECs ameliorates atherosclerosis progression^16^. Several studies implicate MAP4K4 as a proinflammatory kinase that is required to mediate the deleterious functions of TNF-α in multiple cell types^13^,^17^,^18^. Recent reports additionally suggest that endothelial MAP4K4 enhances endothelial permeability, which can promote inflammation and atherosclerosis progression^19^. However, the role of endothelial MAP4K4 in models of *in vivo* chronic inflammatory processes such as atherosclerosis has not yet been assessed. In this study, we observed that MAP4K4 expression and/or activity was increased in the aortas of mice and humans with atherosclerosis. Thus, we hypothesized that endothelial MAP4K4 might play a role in promoting this disease by facilitating endothelial activation in response to inflammatory stimuli.

Here, we present multiple points of evidence that reduction or loss of endothelial MAP4K4 expression profoundly ameliorates atherosclerotic lesion development in mice. Specifically, deletion of Map4k4 in ECs in mice is accompanied by reduced aortic macrophage and chemokine content, reduced EC adhesion molecule expression as well as attenuated leukocyte homing to atherosclerotic plaques. Furthermore, MAP4K4 depletion in ECs lessens inflammatory capacity by reducing TNF-α-mediated permeability, leukocyte adhesion and leukocyte adhesion molecule expression. Finally, treating mice with a novel small-molecule inhibitor with high selectivity for MAP4K4 kinase activity ameliorates atherosclerosis progression and promotes regression in two distinct animal models. Thus, the data presented here indicate a central role for MAP4K4 in promoting vascular inflammation and atherosclerosis.

## Results

### MAP4K4 levels are increased in atherosclerosis

**Figure 1:**
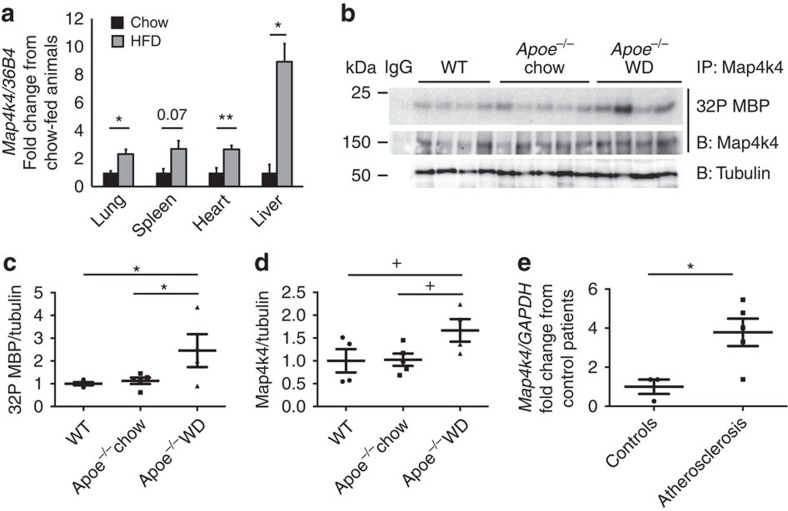
Increased MAP4K4 expression in atherosclerosis. (**a**) Eight-to-ten-week-old mice were fed chow or 60% HFD for 16 weeks, messenger RNA (mRNA) was extracted from the indicated tissues, and quantitative RT–PCR was performed for *Map4k4* and normalized to *36b4.* The data represent the mean±s.e.m. (*P<0.05, **P<0.005, *N*=3–7). (**b**–**d**) Aortas were extracted from age-matched chow-fed wild-type or *Apoe*^−*/*−^ mice or WD-fed *Apoe*^−*/*−^ mice. (**b**) Immune-complex kinase assays were performed in Map4k4 immunoprecipitates using MBP as an exogenous substrate. Lysates were immunoblotted for tubulin as a loading control. (**c**) Densitometric quantification of ^32^P MBP as normalized to tubulin. (**d**) Densitometric quantification of immunoprecipitated Map4k4 as normalized to tubulin (analysis of variance ^+^*P*=0.05, *P<0.05, *N*=4–5). (**e**) mRNA was isolated from normal human arteries or atherosclerotic plaques, and quantitative RT–PCR was performed for *MAP4K4* or *GAPDH* (*P<0.05, *N*=3–5).

To assess whether Map4k4 expression was altered by atherosclerotic risk factors such as obesity, mice were fed a standard chow or 60% high-fat diet (HFD) for 16 weeks, and then several tissues were isolated for quantitative reverse transcription (qRT)–PCR analysis. Interestingly, *Map4k4* messenger RNA (mRNA) expression was increased in several tissues including lung, spleen, heart and liver from HFD-fed mice compared with age-matched, chow-fed counterparts (Fig. 1a).

To assess whether Map4k4 activity and expression was increased in animal models of atherosclerosis, whole aortas were isolated from chow-fed wild-type or *Apoe*^−*/*−^ mice and Western diet (WD)-fed *Apoe*^−*/*−^ mice, and immune-complex protein kinase assays were performed (Fig. 1b). Interestingly, Map4k4 protein kinase activity was increased concomitant with Map4k4 protein expression in WD-fed *Apoe*^−*/*−^ mice compared with chow-fed animals (Fig. 1c–d). Finally, *MAP4K4* mRNA expression was assessed in human atherosclerotic plaques or non-diseased arteries by qRT–PCR. In atherosclerotic plaques, there was a significant 3.8-fold increase in *MAP4K4* mRNA expression compared with coronary or aortic arterial samples from human subjects who did not have atherosclerosis (Fig. 1e). These data are consistent with another recent large-scale human study that identified increased *MAP4K4* gene expression in atherosclerosis^20^. These data provided rationale to examine whether there was a cell-type-specific role for MAP4K4 in atherosclerosis.

### Mice lacking EC MAP4K4 are protected from atherosclerosis

**Figure 2:**
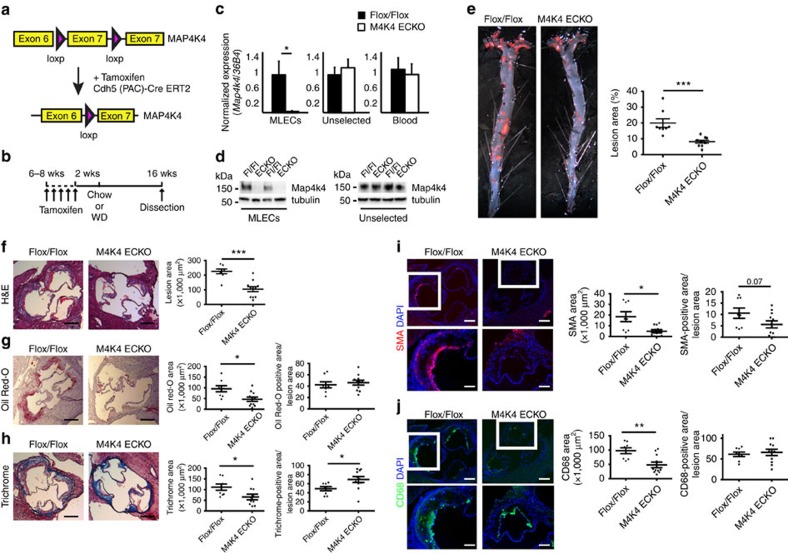
Reduced atherosclerosis in *M4K4* ECKO mice. Map4k4 flox/flox animals were crossed with Cdh5(PAC)-ERT2-Cre animals and injected with tamoxifen for 5 consecutive days at 6–8 weeks of age. (**a**) Cre-mediated Map4k4 exon-7 deletion. (**b**) Schematic of injection and feeding scheme. (**c**) Messenger RNA was extracted and qRT–PCR was performed for *Map4k4* in primary MLECs, the unselected, non-EC fraction of mouse lung cells and peripheral blood leukocytes. The data represent the mean±s.e.m. as normalized to *36b4* expression (*P<0.05, *N*=6–8). (**d**) Immunoblots were performed for Map4k4 or tubulin in immune-selected primary MLECs and the unselected, non-EC fraction of mouse lung cells. Western blots are representative of 6–8 animals per group. (**e**–**j**) Flox/flox and MAP4K4 ECKO mice were crossed with *Apoe*^−*/*−^ mice and fed a WD for 16 weeks as in **b**. (**e**) Left, Oil Red-O-stained *en face* aortic preparations from flox/flox and MAP4K4 ECKO animals. Right, quantification of Oil Red-O-stained area. Data represent the mean±s.e.m. (***P<0.0005, *N*=8–10). (**f**–**j**) Aortic root sections of flox/flox and MAP4K4 ECKO *Apoe*^−*/*−^ animals stained with (**f**) haematoxylin and eosin (H&E) (scale bar, 250 μm), (**g**) Oil Red-O (scale bar, 250 μm), (**h**) trichrome (scale bar, 250 μm), (**i**) smooth muscle actin (scale bar, 250 μm (top image); scale bar, 100 μm (bottom image)) and (**j**) Cd68 (scale bar, 250 μm (top image); scale bar, 100 μm (bottom image)). Left panel, representative images. Right panels, quantification of stained area or as a percentage of lesion area. Data represent the mean±s.e.m. (*P<0.05, **P<0.005, ***P<0.0005, *N*=8–11). DAPI, 4,6-diamidino-2-phenylindole.

Mice lacking MAP4K4 display embryonic lethality^21^. Thus, to assess whether endothelial MAP4K4 contributes to atherosclerosis development, endothelial-specific MAP4K4 knockout mice were generated by crossing MAP4K4 *flox/flox* animals with tamoxifen-inducible VE-cadherin cre mice^22^ (Fig. 2a). At 6–8 weeks of age, MAP4K4 flox/flox (flox/flox) or MAP4K4 flox/flox cre+ (MAP4K4 ECKO) animals were injected with tamoxifen (Fig. 2b). Immune-selected mouse lung ECs (MLECs) derived from MAP4K4 ECKO animals displayed markedly reduced MAP4K4 mRNA and protein expression, whereas cells that were not immune-selected displayed no reduction (Fig. 2c,d). Furthermore, MAP4K4 levels were unaltered in whole-blood leukocytes from these animals, confirming endothelial specificity (Fig. 2d).

MAP4K4 ECKO animals were then crossed with mice on the *Apoe*^−*/*−^ background and fed WD after tamoxifen administration as in Fig. 2b. MAP4K4 ECKO animals demonstrated a 54% reduction in lesion area at the aortic root (226.3 versus 104.9 × 1,000 μm^2^; Fig. 2f), and *en face* preparations revealed that the Oil Red-O-stained area was also reduced by 59% after WD (20.0% versus 8.2%; Fig. 2e). Plaque composition was investigated by staining the aortic roots of flox/flox and MAP4K4 ECKO animals for lipids, collagen, smooth muscle actin and the macrophage marker Cd68. Consistent with the reduction in overall lesion area, reduced staining for all of these parameters was observed in MAP4K4 ECKO aortic roots (Fig. 2g–j). Interestingly, collagen content as a percentage of plaque area was significantly enhanced in MAP4K4 ECKO animals (Fig. 2h). Furthermore, smooth muscle actin staining as a percentage of plaque area demonstrated a trend to reduced area in MAP4K4 ECKO animals (Fig. 2i). These results suggest that consistent with the reduced plaque area, MAP4K4 ECKO mice may also demonstrate less advanced, more stable plaques than control littermates.

**Figure 3:**
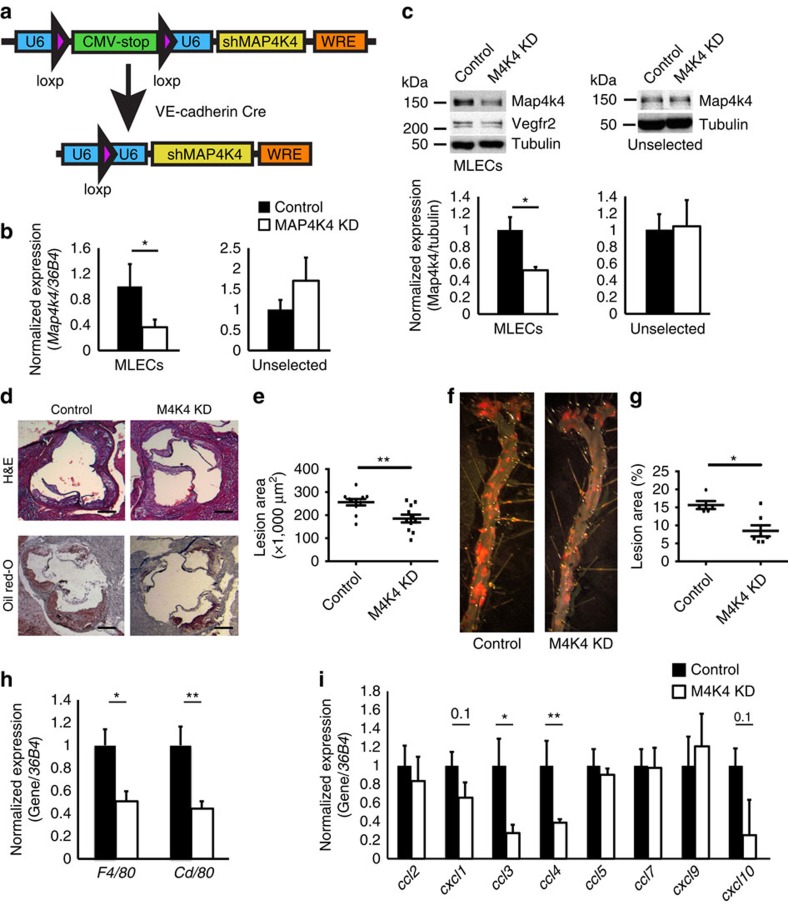
Reduced atherosclerosis in M4K4 KD mice. (**a**) Schematic of the transgenic construct used to generate MAP4K4 KD animals. The U6 promoter becomes reconstituted after cre-mediated recombination to drive tissue-specific shRNA expression. (**b**,**c**) Primary lung endothelial cells (MLECs) were derived from control and MAP4K4 KD animals. (**b**) Messenger RNA (mRNA) was extracted and quantitative RT–PCR was performed for *Map4k4* in immune-selected or unselected cells. The data represent the mean±s.e.m. as normalized to *36b4* expression (*P<0.05, *N*=7). (**c**) Primary endothelial or unselected cell lysates were immunoblotted for Map4k4 and Vegfr2. Data represent the mean±s.e.m. as normalized to tubulin expression (*P<0.05, *N*=3–6). (**d**–**g**) MAP4K4 KD mice were crossed with *Apoe*^−*/*−^ mice and fed a WD for 16 weeks. (**d**) Aortic root sections of control and MAP4K4 KD animals stained with haematoxylin and eosin (H&E) (top) and Oil Red-O (bottom). Scale bar, 250 μm. (**e**) Quantification of aortic root lesion area. Data represent the mean±s.e.m. (**P<0.005, *N*=9,11). (**f**) Oil Red-O-stained *en face* aortic preparations from control and MAP4K4 KD animals. (**g**) Quantification of Oil red-O-stained area. Data represent the mean±s.e.m. (*P<0.05, *N*=5–7). (**h**–**i**) mRNA was prepared from whole aortas, and qPCR was performed. (**h**) Macrophage markers *F4/80* and *Cd68*. (**i**) Chemokines *Ccl2*, *Cxcl1*, *Ccl3*, *Ccl4*, *Ccl5*, *Ccl7*, *Cxcl9* and *Cxcl10*. Data represent the mean±s.e.m. as normalized to *36b4* (*P<0.05, **P<0.005, *N*=9–10).

An additional model of endothelial MAP4K4 deletion was generated in which constitutively expressed cre recombinase expression under control of the VE-cadherin promoter drives endothelial-specific Map4k4 short hairpin RNA (shRNA) expression (Fig. 3a). MLECs from MAP4K4shRNA-VE-cadherin cre-expressing mice (MAP4K4 knock down (KD)) demonstrated a significant reduction, but not total ablation, of Map4k4 mRNA and protein expression compared with the MAP4K4shRNA mice that did not express cre (controls, Fig. 3b,c). MAP4K4 KD mice were crossed onto *Apoe*^−*/*−^ animals, and atherosclerotic lesion size was assessed after WD. MAP4K4 KD animals displayed a significant 31% reduction in aortic root lesion area (256.1 versus 185.1 × 1,000 μm^2^; Fig. 3d,e) and a 46% reduction in *en face* prepared aortic lesion areas as assessed by haematoxylin and eosin staining compared with controls (15.7% versus 8.5%; Fig. 3f,g). These changes in aortic lesion size were in the absence of changes in glucose levels or plasma lipid content (Supplementary Table 1). Importantly, the atherosclerosis protection in these animals was observed even with only a partial loss of endothelial Map4k4 expression. Thus, using both conditional endothelial Map4k4 ablation and constitutive endothelial Map4k4 silencing approaches in mouse genetic models, we demonstrate that endothelial Map4k4 expression is required for full atherosclerotic lesion development and progression.

### Less aortic macrophage content in mice lacking EC Map4k4

**Figure 4:**
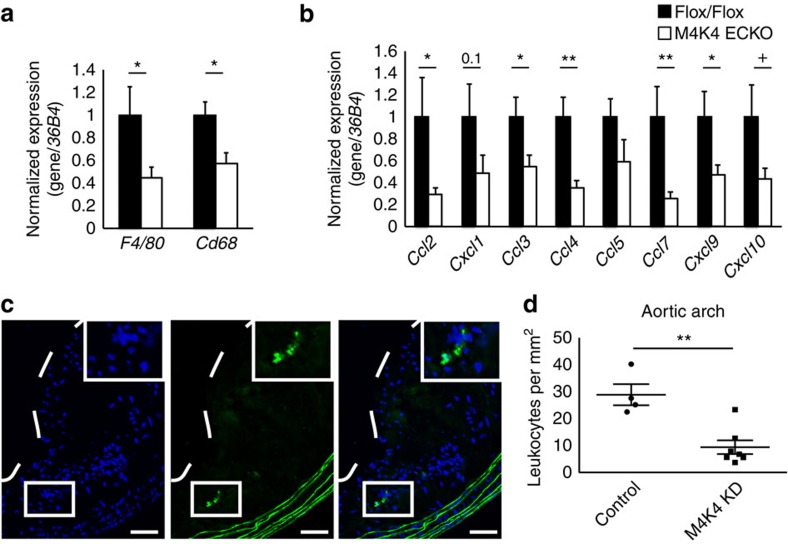
Less macrophages in plaques from M4K4 ECKO and KD mice. (**a**,**b**) Messenger RNA was prepared from whole aortas, and qPCR was performed. (**a**) Macrophage markers *F4/80* and *Cd68*. (**b**) Chemokines *Ccl2*, *Cxcl1*, *Ccl3*, *Ccl4*, *Ccl5*, *Ccl7*, *Cxcl9* and *Cxcl10*. Data represent the mean±s.e.m. as normalized to *36b4* (*P<0.05, **P<0.005, *N*=9–10). (**c**,**d**) Homing of GFP leukocytes into atherosclerotic lesions 48 h after intravenous injection into control or MAP4K4 KD mice that were fed WD for 16 weeks. (**c**) Fluorescence micrograph of atherosclerotic plaque demonstrating four GFP leukocytes within the aortic arch. The dashed line indicates the plaque border. Inset, magnification of three GFP leukocytes. Left, 4,6-diamidino-2-phenylindole; middle, GFP; right, merge. Scale bars, 100 μm. (**d**) Quantification of GFP leukocytes per square millimetre of plaque. Data represent the mean±s.e.m. (**P<0.005, *N*=4,7).

We hypothesized that the protection from atherosclerosis observed in MAP4K4 ECKO and MAP4K4 KD animals was due to impaired leukocyte recruitment through the EC barrier and accumulation within the atherosclerotic plaque. Thus, aortas from control and MAP4K4 ECKO or MAP4K4 KD mice were isolated, and macrophage gene expression was estimated by qRT–PCR. Macrophage marker genes *F4/80* and *Cd68* were significantly reduced in both MAP4K4 ECKO and MAP4K4 KD mice compared with their respective controls (Figs 3h and 4a). Because endothelial-derived chemokines can also control leukocyte homing, we additionally assessed the expression of several chemokines that are known to play a role in atherosclerosis. Analysis by qRT–PCR revealed decreased expression of *Ccl3* and *Ccl4* in MAP4K4 KD mouse aortas compared with controls (Fig. 3i). The reduction in chemokine mRNA levels was more marked in MAP4K4 ECKO mouse aortas; in this model, *Ccl2*, *Ccl7* and *Cxcl9* expression was also reduced (Fig. 4b), which is consistent with our observations that MAP4K4 ECKO mice display a near-complete ablation of endothelial Map4k4 protein levels, whereas MAP4K4 KD mice only display a partial reduction (Figs 2c,d and 3b,c).

**Figure 5:**
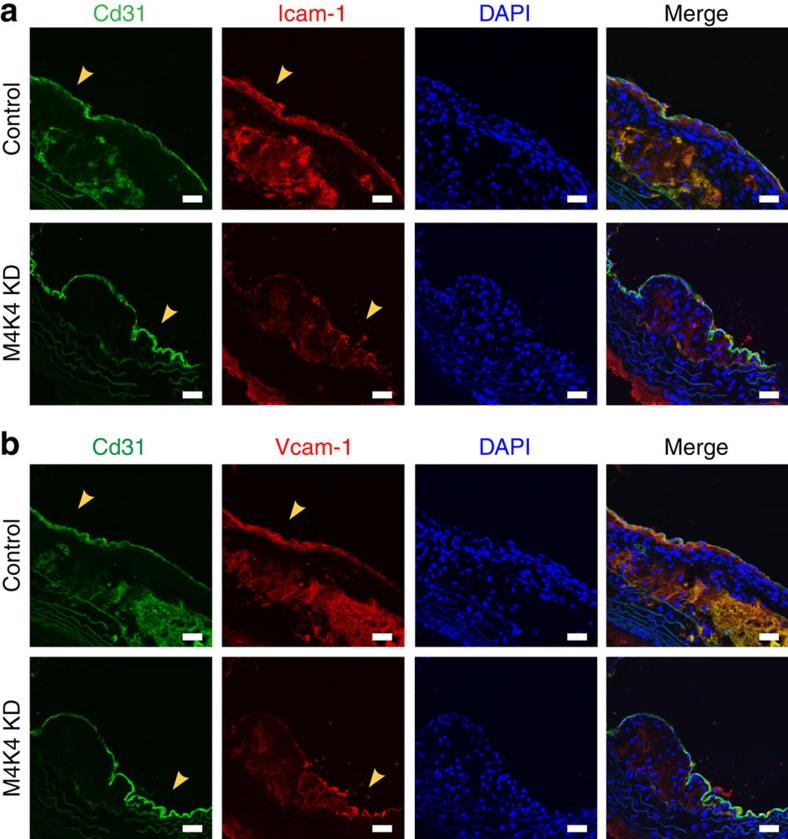
Reduced EC Icam-1 and Vcam-1 in M4K4 KD plaques. (**a**,**b**) Control and M4K4 KD mice were fed WD for 16 weeks. Aortic arch sections were stained for Cd31 as an endothelial marker (green), Icam-1 (red, (**a**)), Vcam-1 (red, (**b**)) or DAPI. From left, CD31, Icam-1/Vcam-1, DAPI and merge. Scale bars, 100 μm. Arrowheads indicate Cd31/Icam-1 or Cd31/Vcam-1 colocalization. Images are representative of at least three control and three M4K4 KD animals. DAPI, 4,6-diamidino-2-phenylindole.

To formally determine whether endothelial MAP4K4 promoted leukocyte recruitment, thioglycollate-elicited peritoneal exudate cells were extracted from green fluorescent protein (GFP)-expressing mice and injected intravenously into control or MAP4K4 KD animals, and GFP-positive cell content was quantified within the aortic arch to assess homing (Fig. 4c). MAP4K4 KD mice displayed a 67% reduction in GFP-positive cell content within atherosclerotic plaques (Fig. 4d). Macrophage homing to inflamed sites is controlled *in vivo* by the expression of leukocyte adhesion molecules Intercellular adhesion molecule (Icam)-1 and Vcam-1. Thus, we assessed expression of these molecules in the aortic arch sections after WD. Immunofluorescence of the aortic arches in control mice revealed significant levels of both Icam-1 and Vcam-1 protein that colocalized with the endothelial marker Cd31, whereas in M4K4 KD aortas, there was little Icam-1 and Vcam-1 staining that colocalized with Cd31 (Fig. 5), suggesting that the reduced macrophage accumulation in the plaques may be due to a reduction in macrophage homing. Thus, endothelial MAP4K4 plays an essential role in leukocyte homing to and accumulation within atherosclerotic plaques.

### EC MAP4K4 regulates TNF-α-mediated inflammation *in vitro*

**Figure 6:**
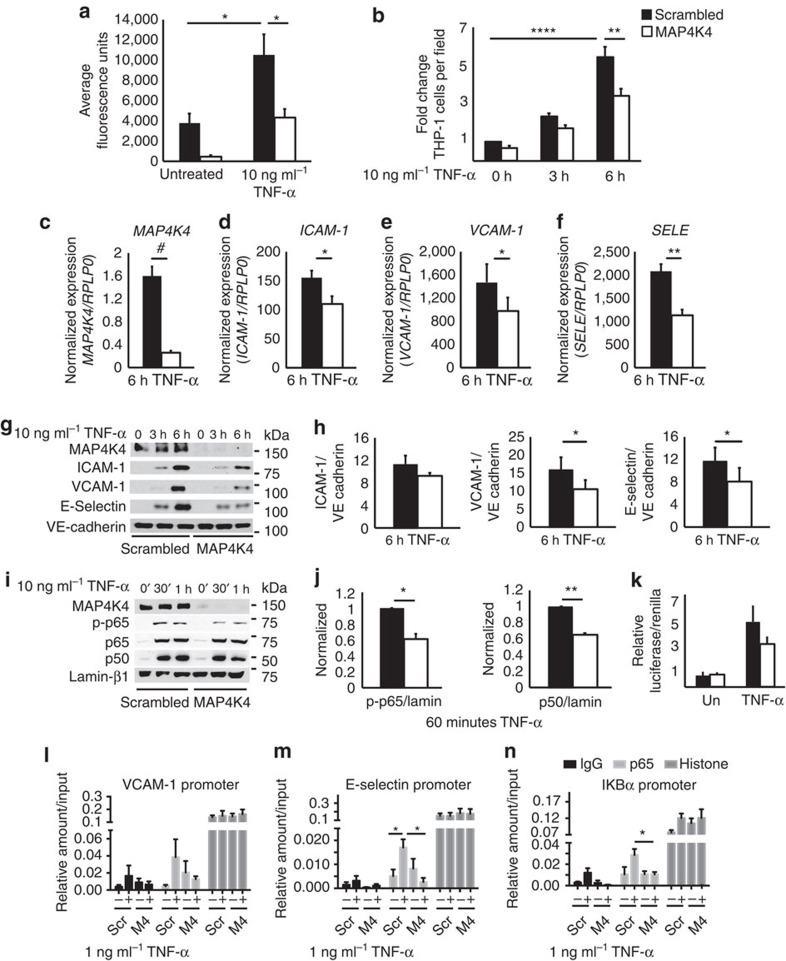
Endothelial MAP4K4 promotes EC activation via NF-κB. HUVECs were treated with scrambled or MAP4K4 siRNA, and cells were stimulated with 1 or 10 ng ml^−1^ TNF-α for the indicated times. (**a**) Cells were seeded onto transwell chambers. Confluent cells were treated overnight with 10 ng ml^−1^ TNF-α or left untreated, and FITC-labelled dextran that migrated through the HUVEC monolayer was measured. The data represent the mean fluorescence intensity±s.e.m. (analysis of variance (ANOVA) *P<0.05, *N*=4). (**b**) THP-1 monocytes were stained with calcein green and adhered to activated endothelium for 30 min. Fluorescence microscopy was performed to determine the number of adherent THP-1 monocytes per microscopic field (× 100). The data represent the mean±s.e.m. as normalized to the unstimulated control time point (ANOVA **P<0.01, ****P<0.0001, *N*=5). (**c**–**f**) Messenger RNA was extracted and quantitative RT–PCR was performed for (**c**) *MAP4K4*, (**d**) *ICAM-1*, (**e**) *VCAM-1* and (**f**) *SELE*. The data represent the mean±s.e.m. as normalized to RPLP0 (*P<0.05, **P<0.005, ^#^*P*<0.0001, *N*=5–7). (**g**) Immunoblots were performed for ICAM-1, VCAM-1 and E-selectin. (**h**) Densitometric analyses from **g**. The data represent the means±s.e.m. as normalized to VE-cadherin (*P<0.05, *N*=4–6). (**i**) Biochemical fractionations were performed, and nuclear fractions were immunoblotted for MAP4K4, p-p65, total p65, p50 and lamin-β1. (**j**) Densitometric analyses represent the mean±s.e.m. for the 60-min time point as normalized to lamin-β1 (*P<0.05, **P<0.005, *N*=3). (**k**) NFκB-luciferase and SV40-Renilla were transfected into HUVECs after treatment with scrambled or MAP4K4 siRNA. Cells were left unstimulated or stimulated with 10 ng ml^−1^ TNF-α overnight before luciferase and Renilla measurement. The data represent the mean±s.e.m. from four independent experiments. (**l**–**n**) HUVECs were transfected with MAP4K4 siRNA and stimulated or not with 1 ng ml^−1^ TNF-α for 1 h. IgG, p65 and histone antibodies were used to immunoprecipitate chromatin and RT–PCR was used to amplify (**l**) E-selectin, (**m**) VCAM-1 and (**n**) IκBα promoters. The data represent the mean±s.e.m. as normalized to input (*P<0.05, *N*=3–4).

The significant reduction in atherosclerosis that was observed in MAP4K4-depleted mice suggested that endothelial MAP4K4 was a potent regulator of vascular inflammation. Thus, we assessed whether MAP4K4 was required for TNF-α-mediated inflammation of the endothelium, including tight-junction permeability, which allows for plasma protein leakage into inflamed tissues^23^ and inflammatory cell adhesion to activated endothelium. For these experiments, human umbilical vein ECs (HUVECs) were employed as a standard model for such analysis. Using transwell chambers, it was observed that TNF-α induced a 65% increase in FITC-labelled dextran movement across a monolayer in scrambled short interfering RNA (siRNA)-transfected HUVECs, whereas permeability was 87% and 59% reduced in MAP4K4-silenced HUVECs, basally and after stimulation, respectively (Fig. 6a). MAP4K4 also promoted monocyte adhesion to an activated endothelial monolayer. Though TNF-α treatment increased monocyte adhesion to scrambled siRNA-transfected HUVECs by 65%, both basal and stimulated monocyte adhesion to the endothelium was reduced by 31 and 37%, respectively, after MAP4K4 silencing (Fig. 6b).

Because MAP4K4 promoted leukocyte adhesion to HUVECs, we assessed whether MAP4K4 was required for TNF-α-induced cell surface adhesion molecule upregulation. Upon MAP4K4 silencing with siRNA, ICAM-1, VCAM-1 and E-selectin mRNA and protein expression was blunted compared with scrambled siRNA-transfected cells at multiple time points (Fig. 6c–h; Supplementary Fig. 1a–d). MLECs from MAP4K4 KD mice also displayed attenuated TNF-α-induced leukocyte adhesion molecule expression compared with those from controls (Supplementary Fig. 1e–h). These data indicate that endothelial MAP4K4 plays a cell autonomous and conserved role among species to regulate TNF-α-induced leukocyte adhesion molecule expression. Similar experiments were conducted using other inflammatory stimuli in HUVECs. Although a similar trend to reduced E-selectin mRNA expression was observed in response to interleukin-1β treatment after MAP4K4 silencing (Supplementary Fig. 1i–l), no changes were observed after lipopolysaccharide treatment (Supplementary Fig. 1m–p), suggesting some specificity to the actions of MAP4K4 to mediate vascular inflammation.

### Endothelial MAP4K4 promotes NFKB localization and activity

EC leukocyte adhesion molecule expression is controlled by the MAPK and NFκB signalling pathways^15^,^24^,^25^,^26^,^27^. MAP4K4 was originally reported to act upstream of the JNK signalling cascade^28^,^29^; however, no reductions in TNF-α-induced JNK, p38 MAPK or extracellular signal-regulated kinase (Erk) activation were noted upon MAP4K4 depletion (Supplementary Fig. 2a), consistent with recent reports demonstrating no requirement for MAP4K4 in JNK activation^12^,^13^,^14^. Because of the strong link between endothelial NFκB and atherosclerosis^16^,^30^,^31^, we thus investigated whether MAP4K4 might promote NFκB activation. It is well established that p65 (Rel A) is retained in the cytosol by inhibitor of κBα (IκBα), which is phosphorylated and subsequently degraded upon stimulation with cytokines, thus allowing p65 nuclear translocation and target gene expression^32^. However, neither TNF-α-mediated IκBα phosphorylation nor its degradation were altered (Supplementary Fig. 2b–e) after MAP4K4 silencing. Furthermore, IκB kinase and p65 phosphorylation was also unaltered (Supplementary Fig. 2b). However, in the basal state, total IκBα protein levels were increased in MAP4K4-deficient HUVECs compared with control cells (Supplementary Fig. 2b,c).

The lack of changes due to MAP4K4 depletion in TNF-α-mediated IκB kinase or IκBα phosphorylation did not exclude the possibility that MAP4K4 could regulate nuclear p65 levels or activity. Thus, MAP4K4 or scrambled siRNA-transfected HUVECs were subjected to biochemical fractionation. In MAP4K4-depleted cells, less nuclear phospho-p65 and its heterodimeric partner p50 protein was observed after TNF-α stimulation compared with scrambled-transfected cells (Fig. 6i,j). These data suggested that MAP4K4 might regulate NFκB nuclear retention or activity. Indeed, TNF-α-induced NFκB transcriptional activity, as assessed by a luciferase reporter containing NFκB-binding elements^33^, was attenuated when MAP4K4 was silenced (Fig. 6k). Furthermore, using p65 chromatin immunoprecipitation in HUVECs, TNF-α-induced p65 binding to the VCAM-1, E-selectin and IKBα promoters (Fig. 6l–n) was reduced in MAP4K4-silenced cells, suggesting that MAP4K4 may regulate NFκB target gene expression.

### Pharmacological MAP4K4 inhibition reduced atherosclerosis

On the basis of the protection observed in development of atherosclerosis after MAP4K4 depletion in genetic mouse models (Figs 2 and 3), we attempted to verify these results and determine whether inhibiting the protein kinase activity of MAP4K4 would also protect against atherosclerosis development. A highly selective small-molecule inhibitor of MAP4K4, PF-06260933, was therefore employed, which is described in detail elsewhere^34^. HUVECs or peritoneal macrophages were treated with vehicle or PF-06260933 *in vitro* to determine whether pharmacological inhibition of MAP4K4 altered MAPK signalling in response to TNF-α. No changes were noted in TNF-α-induced JNK, p38 MAPK or Erk phosphorylation in response to inhibitor treatment (Supplementary Fig. 3a–d). PF-06260933 treatment of human aortic ECs also robustly prevented TNF-α-mediated endothelial permeability *in vitro*, similar to MAP4K4 knockdown (Supplementary Fig. 3e).

**Figure 7:**
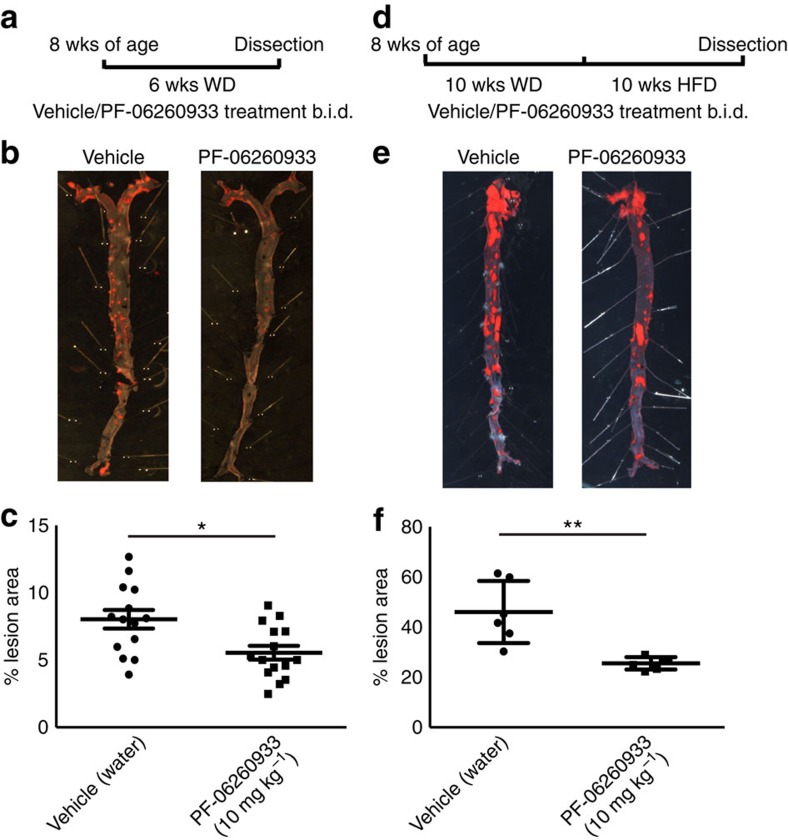
Pharmacological MAP4K4 inhibition ameliorates atherosclerosis. (**a**–**c**) *ApoE*^−*/*−^ mice were administered PF-06260933 or vehicle twice daily for 6 weeks. (**a**) Dosing regimen (**b**) Oil red-O-stained *en face* aortic preparations from vehicle and PF-06260933-treated animals. (**c**) Quantification of the Oil Red-O-stained area. Data represent the mean±s.e.m. (*P<0.05, *N*=14–15). (**d**–**f**) *Ldlr*^−*/*−^ mice were fed HFD for 10 weeks followed by PF-06260933 or vehicle administration daily for 10 additional weeks. (**d**) Dosing regimen. (**e**) Oil Red-O-stained *en face* aortic preparations from vehicle and PF-06260933-treated animals. (**f**) Quantification of the Oil Red-O-stained area. Data represent the mean±s.e.m. (**P<0.005, *N*=6).

PF-06260933 was administered to *Apoe*^−*/*−^ mice (10 mg kg^−1^ b.i.d.) for 6 weeks concomitant with WD, and atherosclerosis lesion development was markedly reduced in the treated mice compared with those that were administered vehicle only (8.0% versus 5.5%; Fig. 7a–c). In this animal model, PF-06260933 treatment did not alter plasma lipid content, although reductions in glucose levels were observed (Supplementary Table 1), which is consistent with whole-body-inducible Map4k4 knockout animals^35^. Because atherosclerosis is often treated as an advanced disease, a regression model was also performed with PF-06260933 treatment of *Ldlr*^−*/*−^ mice that were fed a custom diet containing a high cholesterol content. PF-06260933 administration ameliorated further plaque development and/or promoted plaque regression in this animal model (46.0% versus 25.5%; Fig. 7d–f), and a reduction in plasma glucose as well as lipid content was also observed (Supplementary Table 1). Importantly, PF-06260933 administration did not significantly affect body weight in either the *Apoe*^−*/*−^ or *Ldlr*^−*/*−^ mouse models (PF-06260933 serum concentrations were 2275.0±270.1 ng ml^−1^; Supplementary Table 1). These data suggest that PF-06260933 ameliorated atherosclerotic plaque development in animals and may have additional beneficial functions that may be related to effects in non-EC types to reduce plasma glucose levels and lipid content. Thus, both genetic and pharmacological targeting approaches indicate that endothelial MAP4K4, by controlling immune cell recruitment, vascular permeability or both, is required for atherosclerotic plaque development *in vivo*.

## Discussion

Atherosclerosis is an inflammatory disease that is initiated by lipid-mediated vascular inflammation of the vessel wall, which promotes continual monocyte recruitment in a leukocyte adhesion molecule-dependent manner^4^,^8^,^36^. Strategies for developing therapeutics for this disease include the search for key cellular enzymes in ECs that promote leukocyte attachment and permeability and may serve as drug targets. We hypothesized that the protein kinase MAP4K4 may be a candidate based on previous work suggesting it promotes proinflammatory functions, and the data presented here support this hypothesis. Constitutive silencing of endothelial MAP4K4 or inducible endothelial-specific MAP4K4 deletion ameliorated atherosclerosis development in mice as measured by histology of the aortic root and Oil Red-O staining of the aorta (Figs 2 and 3). Furthermore, reduced macrophage marker and chemokine mRNA expression was observed in aortas from *Apoe*^−*/*−^ animals when endothelial MAP4K4 expression was attenuated, and leukocyte homing to atherosclerotic plaques within the aortic arch was also markedly inhibited (Fig. 4). Finally, treatment of *Apoe*^−*/*−^ or *Ldlr*^−*/*−^ mice with a small-molecule MAP4K4 protein kinase inhibitor significantly reduced atherosclerotic plaque progression and promoted plaque regression (Fig. 7). Together, these data strongly support the idea that MAP4K4 may be a novel therapeutic target for the treatment of cardiovascular disease.

To understand the mechanisms by which MAP4K4 promoted monocyte accumulation in atherosclerotic plaques, we utilized human ECs as a model system. With both HUVECs and human aortic ECs, the results showed that MAP4K4 silencing and pharmacological inhibition blunted basal and TNF-α-mediated endothelial permeability (Fig. 6; Supplementary Fig. 3). Furthermore, siRNA-mediated MAP4K4 depletion in HUVECs attenuated TNF-α-induced leukocyte adhesion, which was accompanied by a reduction in TNF-α-induced adhesion molecule expression. Finally, less NFκB p65 binding to adhesion molecule promoters was observed in TNF-α-treated MAP4K4-depleted cells, indicating that endothelial MAP4K4 promotes NFκB-mediated gene expression (Fig. 6).

We previously demonstrated that macrophage MAP4K4 was not upstream of the classical activation system for the NFκB signalling pathway^13^; however, in the present experiments we noted that MAP4K4 silencing reduced TNF-α-induced p65 nuclear localization and transcriptional activation without altering IκBα degradation (Fig. 6i,j; Supplementary Fig. 2). These data suggest an alternative mechanism of NFκB modulation by MAP4K4, which was not addressed in our studies on macrophages. The reduced transcriptional activation and promoter binding in response to MAP4K4 depletion observed in these present studies may be due to altered post-translational modifications of p65 or p50, such as acetylation that can control p65 activity and nuclear retention in a manner downstream of IκBα degradation^37^. Future studies will assess this possibility.

The previous demonstration that MAP4K4 is required for inflammatory cytokine release in a model of lipopolysaccharide-induced lethality^13^ has been confirmed using PF-06260933 (ref. 34). These results suggest that macrophage MAP4K4 can modulate cytokine release, and macrophage-derived cytokines can contribute to atherosclerosis progression in mice and humans^1^. Thus, loss of MAP4K4 expression or activity in macrophages using genetic deletion strategies or via PF-06260933 treatment, respectively, could also ameliorate atherosclerosis. Genetic tissue-specific deletions of MAP4K4 in macrophages as well as smooth muscle on the *Apoe*^−*/*−^ background will be performed in the future to assess the contribution of these tissues to the overall atherosclerosis protection observed after PF-06260933 treatment.

It is important to emphasize that neither endothelial MAP4K4 silencing nor pharmacological inhibition of MAP4K4 in ECs or peritoneal macrophages altered TNF-α-mediated JNK, p38 MAPK or Erk activation (Supplementary Figs 2 and 3). These data are consistent with animal models that illustrate no requirement for JNK1 in atherosclerosis^38^. Although no endothelial-specific JNK2 knockout mice have been reported, the atherosclerosis protection mediated by JNK2 deletion is presumably because of enhanced macrophage lipid flux^38^,^39^. Furthermore, endothelial NFκB is required for leukocyte adhesion molecule expression, atherosclerosis and macrophage homing to atherosclerotic plaques^16^,^30^, and pharmacological targeting of endothelial NFκB ameliorated leukocyte–endothelial interactions as well as vascular permeability^31^. In addition to the inflammatory response, endothelial NFκB also regulates systemic insulin sensitivity and even ageing^31^,^40^.

Notably, PF-06260933 treatment of *Apoe*^−*/*−^ and *Ldlr*^−*/*−^ animals reduced plasma glucose levels, indicating that systemic MAP4K4 inhibition with this compound may also enhance peripheral insulin sensitivity and have anti-diabetic actions. In support of this hypothesis, a recent publication from our laboratory describes whole-body-inducible and myf5-specific Map4k4 knockout mice, which display reduced plasma glucose levels and enhanced insulin sensitivity after HFD compared with control littermates^35^. Though there were no significant differences in plasma lipid content in either MAP4K4 KD mice or PF-06260933-treated *Apoe*^−*/*−^ mice, there were significant reductions in PF-06260933-treated *Ldlr*^−*/*−^ animals, which could contribute to the plaque size changes observed in this model (Supplementary Table 1). Endothelial Map4k4 KD mice did not display altered plasma lipid levels (Supplementary Table 1); therefore, the reduction in plasma lipids that was observed in *Ldlr*^−*/*−^ mice after PF-06260933 treatment could contribute to the reduced atherosclerosis in a manner that is independent of the endothelium. However, the *Ldlr*^−*/*−^ mouse study was performed using a diet that was extremely high in cholesterol content (1.25%) and represents an extreme dietary stress, and *Apoe*^−*/*−^ studies were performed using a diet with much lower cholesterol content (0.2%).

Although we have focused here on the role of endothelial MAP4K4 using genetic models, we cannot rule out the possibility that systemic MAP4K4 inhibition with PF-06260933 may also ameliorate atherosclerosis via its effects on additional cell types such as macrophages, smooth muscle cells or even the liver. Therefore, while MAP4K4 likely has actions in multiple cell types to promote atherosclerosis in mice, protection from and treatment of atherosclerosis by PF-06260933 is consistent with endothelial MAP4K4 as one of multiple targets for the action of PF-06260933 to mediate these beneficial effects.

In summary, our data generated from the methods of gene silencing, gene ablation and pharmacological inhibition identify endothelial MAP4K4 as a novel regulator of vascular inflammation and endothelial activation that is required for atherosclerotic plaque development in mice. These data also implicate endothelial MAP4K4 in acute endothelial functions that are impaired by inflammation such as endothelial barrier function or relaxation. Furthermore, the results we present with specific MAP4K4 kinase inhibitor PF-06260933 reveals that MAP4K4 is a novel and important anti-atherosclerosis therapeutic target.

## Methods

### Mouse models

The University of Massachusetts Medical School Institutional Animal Care and Use Committee or the Pfizer Institutional Animal Care and Use Committees approved all of the animal procedures. MAP4K4-pSico mice^41^ were first crossed with VE-cadherin Cre transgenic mice (B6.Cg-Tg(Cdh5-cre)7Mlia/J, Jackson Laboratories), and these animals were then crossed onto the *Apoe*^−*/*−^ background (B6.129P2-*Apoe*^*tm1Unc*^/J, Jackson Laboratories). Map4k4 flox/flox animals were crossed with a tamoxifen-inducible endothelial-specific cre mouse line (Cdh5(PAC)-CreERT2) that was obtained from Dr Ralf Adams^22^, and these animals were crossed onto the *ApoE−/−* background as above.

### Atherosclerosis models

At 6–8 weeks of age, male flox/flox and flox/flox/cre+ littermates were injected with 1 mg tamoxifen per day in corn oil (Sigma) for 5 days. At 5–6 weeks of age (KD mice) or 2 weeks after tamoxifen injection (flox mice), the mice were fed chow or WD (0.2% cholesterol, TD 88137, Harlan Laboratories) for 16 weeks. Compound PF-06260933 Sigma Aldrich (catalog # PZ0272) (10 mg kg^−1^, dissolved in dH_2_O) was orally administered to 8–10-week-old male *ApoE*^−*/*−^ mice twice daily for 6 weeks. *Ldlr*^−*/*−^ male mice (B6.129S7-*Ldlr*^*tm1Her*^/J, Jackson Laboratories, 8–10 weeks old) were placed on HFD (1.25% cholesterol, TD96121, Harlan Laboratories) for 10 weeks before drug administration. Compound PF-06260933 was administered to male 8–10-week-old *Ldlr*^−*/*−^ mice as above for 10 weeks. Oral administration of water was used as vehicle control in all studies. Mice were euthanized by CO_2_ inhalation followed by bilateral pneumothorax. No statistical methods were used to predict sample size, no randomization was performed and the investigations were not blinded during the knockout animal analyses, but were blinded during the drug treatment analyses.

### Generation of mice

The original pSico-MAP4K4 lentiviral vector from the study by Ventura *et al*.^41^ was modified by cleavage to remove viral elements (5′ SIN-LTR, HIV-RRE, flap and 3′ SIN-LTR), which left only the U6 promoter, first loxP site, cytomegalovirus (CMV)-enhanced GFP (EGFP) cassette, second loxP site, MAP4K4 shRNA-coding region, and Woodchuck hepatitis virus post-transcriptional regulatory element. To create a conditional U6 promoter, a CMV-enhanced stop cassette was inserted between two loxP sites. A functional U6 promoter is generated after cre excision, which drives expression of a hairpin targeting MAP4K4 (5′-GCTGTCTGGTGAAGAATTA-3′). Because the polyA tail that is necessary for EGFP expression is in the 3′ SIN-LTR, we were able to preclude any possibility of EGFP expression in primary tissues and thus any side effects of EGFP expression. The U6 promoter still produced MAP4K4 siRNA transcripts, because the 6-nucleotide polyT sequence at the end of the MAP4K4 shRNA antisense sequence is recognized as a termination signal by RNA pol III promoters including U6. The construct was injected into eggs at the one-cell stage, and two-cell stage eggs were implanted into pseudo-pregnant C57Bl/6 J females by the UMASS transgenic animal facility. The animals were then backcrossed onto C57Bl6/J mice for seven generations. Genomic DNA was extracted from the obtained mice and subjected to PCR for genotyping (shRNA primer 5′-CCCGTATGGCTTTCATTTTCTCC-3′, 5′-AAGGAAGGTCCGCTGGATTGAG-3′). Cre and ApoE genotyping was performed according to the method of Jackson Laboratories. MAP4K4 flox/flox animals were obtained from the Texas A&M Institute for genomic medicine. Animals were backcrossed with BL6/J mice for seven generations before crossing them with the Cdh5(PAC)-CreERT2 mouse line. For Map4k4 expression studies, male MAP4K4 flox/flox animals (without cre) were fed standard chow or HFD (60%; RD12942i Research diets) starting 2 weeks after tamoxifen injections, and tissues were analysed after 16 weeks on the respective diet. Mice were fasted overnight before plasma lipid and glucose analysis.

### Human samples

Samples of atherosclerotic plaque were obtained from four patients undergoing elective carotid endarterectomy for significant carotid stenosis. The study was approved by the UMASS Medical School Institutional Review Board (#H00001329), and written informed consent was obtained from each patient before the study. The entire plaque was removed at the time of surgery and immediately frozen in liquid nitrogen. Patient clinical information is listed in Supplementary Table 3. Normal human arterial RNA samples were purchased from Agilent (540199), Clontech (636546), and Biochain (R1234013-10). One additional human atherosclerotic plaque RNA sample was purchased from Origene (CR562657).

### Cell culture and transfection

HUVECs (purchased from Clonetics, mycoplasma negative, C2519AS) were maintained in EGM2 media (Lonza) at 37 °C and 5% CO_2_. Scrambled (5′-GACCAACUCUGGCUUGUUA-3′) or MAP4K4 (5′-CAGUCGCGUUUGCGACUGG-3′) siRNA (25 nM, Dharmacon) was reverse transfected with RNAiMax (Life Technologies) for 48 h before experiments.

### RNA isolation and qRT–PCR

Total RNA was isolated following the manufacturer's protocol (TriPure, Roche). Precipitated RNA was treated with DNAse (DNA-free, Life Technologies) before reverse transcription (iScript Reverse transcriptase, Bio-Rad). SYBR green quantitative PCR (iQ SYBR green supermix, Bio-Rad) was performed on the Bio-Rad CFX97. Primer sequences are listed in Supplementary Table 2.

### Western blotting

Cells were lysed in RIPA lysis buffer (1% NP-40, 50 mM Tris pH 7.4, 150 mM NaCl, 0.1% SDS, 1% sodium deoxycholate and 50 mM EDTA) with 1 × HALT protease and phosphatase inhibitors (Thermo Scientific). Lysates were run on SDS–polyacrylamide gel electrophoresis gels and transferred to nitrocellulose membranes. Membranes were immunoblotted with anti-ICAM-1 BBIG-I1 (1:1,000), VCAM-1 BBIG-V1 (1:1,000), E-selectin BBIG-E4 (1:1,000) (R&D systems), MAP4K4 A301-503A (1:2,000) (Bethyl), VE-cadherin 2500 (1:2,000), p-JNK1/2 4668 (1:1,000), total JNK1/2 9258 (1:1,000), p-p38 MAPK 4631 (1:1,000), p-IκBα 9246 (1:1,000), IκBα 4814 (1:1,000), p-p65 3039 (1:1,000), p65 8242 (1:1,000), HGK 3485 (1:1,000), p-Erk 4370 (1:1,000) (Cell Signaling Technology), total p38 MAPK sc-535 (1:1,000), total Erk1 sc-93 (1:4,000) (Santa Cruz) or lamin-β1 ab16048 (1:1,000) (Abcam) antibodies. Uncropped western blot images are provided as Supplementary Figs 4–6.

### Aortic staining

Mouse aortas were perfused with PBS followed by 10% formalin or 4% paraformaldehyde. Aortic root sections were embedded in OCT for frozen sectioning. Photos of aortic root sections were taken with an Axiovert 35 Zeiss microscope (Zeiss, Germany) equipped with an Axiocam CCl camera at × 25 magnification. *En face* stained aortas and light microscopy was obtained with a Nikon Stereo microscope equipped with a Spot Insight camera (Spot Imaging) at × 5 or × 10 magnification. Aortic root sections and *en face* preparations were stained with Oil Red-O in 60% isopropanol or 80% methanol. Aortic roots were additionally stained with haematoxylin and eosin or trichrome by the UMASS morphology core, with rat anti-mouse CD68 (1:200) (AbD Serotec, clone FA-11) and Cy3-smooth muscle actin (Sigma S6198) (1:200), or with anti-ICAM-1 BBIG-I1 (1:200) or VCAM-1 BBIG-V1 (1:200) (R&D systems) and rat anti-mouse CD31 (1:400) (BD) and mounted in Prolong Gold with DAPI (Life Technologies). Images were quantified using ImageJ or Image Pro Plus Analysis Software (Media Cybernetics).

### Leukocyte homing

Peritoneal exudate cells were elicited by an intraperitoneal injection of 4% thioglycollate into 8–10-week-old male C57BL/6-Tg(UBC-GFP)30Scha/J mice (Jackson Laboratories). After 2 days, 3 million cells were injected intravenously into control or MAP4K4 KD mice that had been fed WD for 16 weeks. After 2 days, aortic arches were embedded in OCT. Total fluorescent cells were counted in 10 × 8-μm sections over a 0.5-mm area, and the cell number was normalized to plaque area.

### Plasma lipid and glucose analysis

Mice were fasted overnight and heparin or EDTA anticoagulated plasma was collected via cardiac puncture. Total cholesterol (Pointe Scientific), total triglyceride and high-density lipoprotein and low-density lipoprotein cholesterol (Sigma) levels were measured using colorimetric assays. Blood glucose levels were measured from whole blood using the Ascensia Breeze glucose analyzer (Bayer). Pfizer lipid studies were analysed with a Clinical Analyzer (C311 Roche/Hitachi).

### THP-1 adhesion

HUVECs were transfected with scrambled or MAP4K4 siRNA as above. THP-1 monocytes were cultured in RPMI media and fluorescently labelled with calcein-AM (BD) according to the package insert 30 min before the adhesion assay. HUVECs were stimulated with 10 ng ml^−1^ TNF-α (Calbiochem) for 3–6 h. In total, 1 × 10^6^ THP-1 cells were incubated with the activated HUVECs for 30 min at 37 °C. Non-adherent THP-1 cells were washed away, the cells were fixed and the remaining cells were imaged with fluorescence microscopy at 488 nm. At least five random fields were quantified per condition as the average number of adherent cells per field.

### Endothelial permeability

The *In Vitro* Vascular Permeability Assay Kit (Millipore) was used according to the manufacturer's instructions. After the cells reached confluence, they were treated overnight with 10 or 100 ng ml^−1^ TNF-α or left untreated.

### Primary EC isolation

Lungs were excised, minced and digested in 0.1% collagenase (Sigma) in RPMI (Life Technologies) for 1 h. Digested cells were filtered, spun and plated in pre-isolation media consisting of 20% heat-inactivated FBS, 40% F-12 HAM media, 40% GlutaMAX media, 2 × L-glutamine, 1 × penicillin/streptomycin (Life Technologies), 0.1 mg ml^−1^ heparin (Sigma), 1 × EC growth supplement (BD). When confluent, cells were magnetically selected with anti-rat dynal beads (Life Technologies) that had been conjugated to anti-CD31 (BD 553369; 10 μg) and anti-CD102 antibodies (BD 553325; 10 μg). Selected cells were grown in EGM2-MV, 15% FBS and 2 × L-glutamine until confluent. Unselected cells were kept as the non-EC fraction and grown until confluent.

### Biochemical fractionation

The NE-PER cellular nuclear fractionation kit (Thermo Scientific) was used for nuclear extract preparation according to manufacturer's instructions.

### Luciferase assay

The NFκB luciferase reporter plasmid was a kind gift from the Sankar Ghosh lab. Luciferase reporter plasmids or control plasmids were transfected into HUVECs with the JET-PEI HUVEC transfection system (Polyplus). After 24 h, cells were treated with 10 ng ml^−1^ TNFα or left untreated for an additional 24 h. Luciferase and Renilla were measured using the dual-luciferase reporter assay system (Promega) according to the manufacturer's instructions.

### Chromatin immunoprecipitation

HUVECs were transfected as described above and either left unstimulated or stimulated with 1 ng ml^−1^ TNFα for 1 h. The Simplechip enzymatic chromatin IP kit (Cell Signaling Technology) was used according to manufacturer's instructions. p65 antibodies (Santa Cruz sc-372; 5 μg) or IgG (Cell Signaling 2729; 5 μg) were used for p65 immunoprecipitations. VCAM-1 and E-selectin (SELE) primer sequences are listed in Supplementary Table 2. IKBα primers were purchased from Cell Signaling Technology.

### Kinase assay

Aortas were lysed in 1% NP-40, 50 mM Tris pH 7.4, 150 mM NaCl, 50 mM EDTA with 1 × HALT protease and phosphatase inhibitors (Thermo Scientific) and immunoprecipitated with Bethyl MAP4K4 antibodies (503 A; 1 μg) or normal rabbit IgG (Cell Signaling 2729; 1 μg). Myelin basic protein (MBP) (1 μg) and 10 μCi of [γ-^32^P]ATP were added to the immunoprecipitates and incubated for 30 min at 30 °C in kinase buffer (20 mM HEPES, 10 nM MgCl_2_, 1 mM dithiothreitol (DTT) and protease and phosphatase inhibitor cocktail). Samples were separated by 12% SDS–polyacrylamide gel electrophoresis and visualized by autoradiography.

### Statistical analysis

A two-tailed Student's *t*-test was used to compare two groups in Microsoft Excel. Where indicated, experiments comparing multiple groups were analysed with one-way analysis of variance and Fisher's exact test or Bonferroni's post-test in Graph Pad Prism 6.0. *P*<0.05 was considered to be statistically significant. Variance was estimated using the s.e.m.

## Additional information

**How to cite this article:** Roth Flach, R.J. *et al*. Endothelial protein kinase MAP4K4 promotes vascular inflammation and atherosclerosis. *Nat. Commun.* 6:8995 doi: 10.1038/ncomms9995 (2015).

## Supplementary Material

Supplementary Information Supplementary Figures 1-6 and Supplementary Tables 1-3
