# Supplementary material for: Endothelial protein kinase MAP4K4 promotes vascular inflammation and atherosclerosis
Source: Nat Commun. 2015 Dec 21;6:8995. doi: 10.1038/ncomms9995 (PMC4703891; doi:10.1038/ncomms9995)
Supplement: Supplementary Information — Supplementary Figures 1-6 and Supplementary Tables 1-3 [file ncomms9995-s1.pdf]

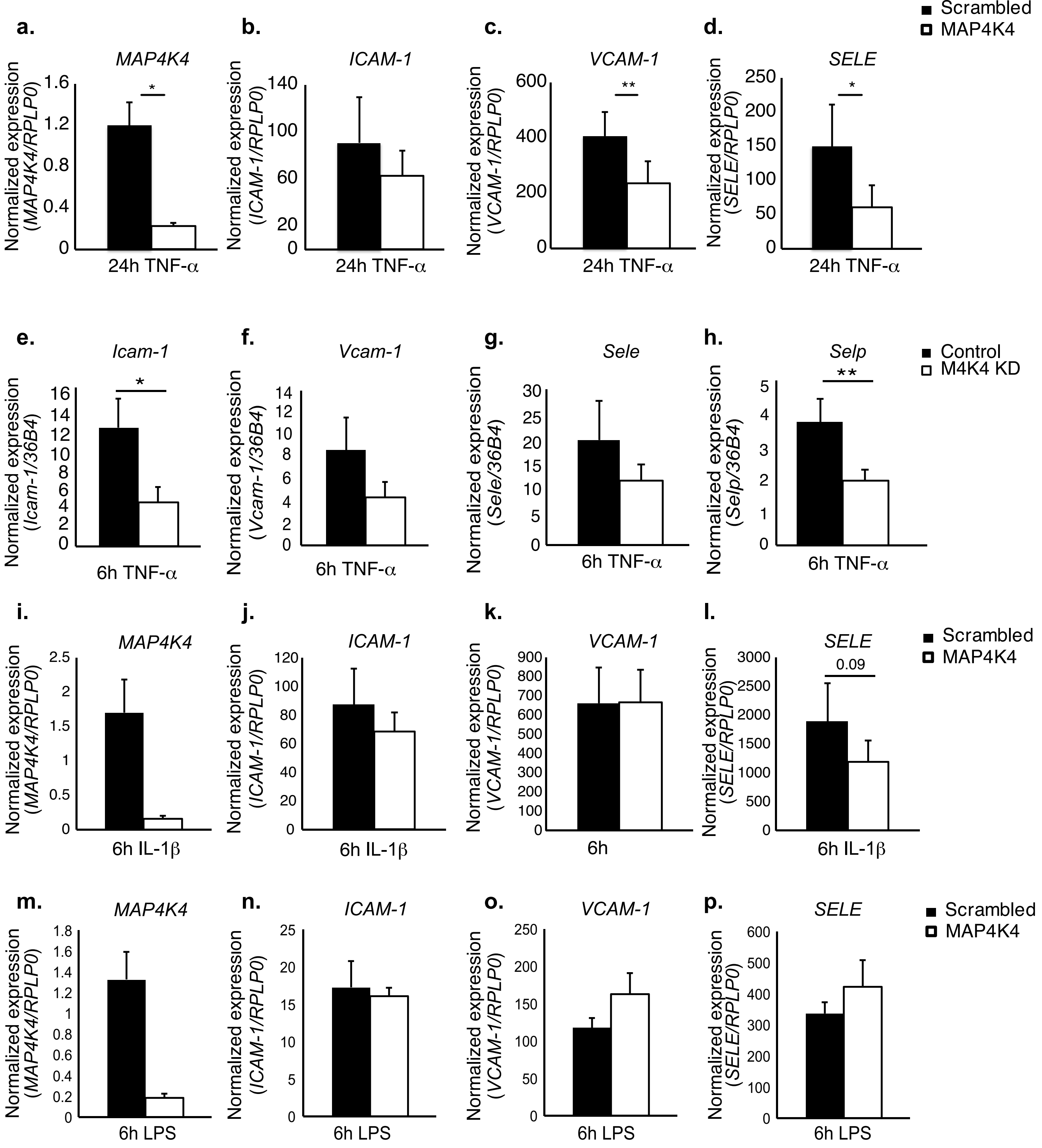

Supplementary Figure 1. Reduced adhesion molecule expression after MAP4K4 silencing. a-d. HUVECs were treated with scrambled or MAP4K4 siRNA and stimulated with 10 ng/mL TNF-α for 24h. RNA was extracted, and qPCR was performed for **a.** *MAP4K4*, **b.** *ICAM-1*, **c.** *VCAM-1*, **d.** *SELE*. The data represent the mean ± SEM as normalized to *RPLP0* (*; p<0.05, **; p<0.005, N=5-7). **e-h.** Primary MLECs were stimulated with 10 ng/mL TNF-α for 6h or left unstimulated, RNA was extracted, and qPCR was performed. **e.** *Icam-1*, **f.** *Vcam-1*, **g.** *Sele*, **h.** *Selp*. Data represent the mean ± SEM as normalized to *36b4*. (ANOVA *; p<0.05, **; p<0.01, N=7-9). **i-l.** HUVECs were treated with scrambled or MAP4K4 siRNA and stimulated with 2 ng/mL IL-1β for 6h. RNA was extracted, and qPCR was performed for **i.** *MAP4K4*, **j.** *ICAM-1*, **k.** *VCAM-1*, **l.** *SELE*. The data represent the mean ± SEM as normalized to *RPLP0* (N=4). **m-p.** HUVECs were treated with scrambled or MAP4K4 siRNA and stimulated with 1 ug/mL LPS for 6h. RNA was extracted, and qPCR was performed for **m.** *MAP4K4*, **n.** *ICAM-1*, **o.** *VCAM-1*, **p.** *SELE*. The data represent the mean ± SEM as normalized to *RPLP0* (N=4-5).

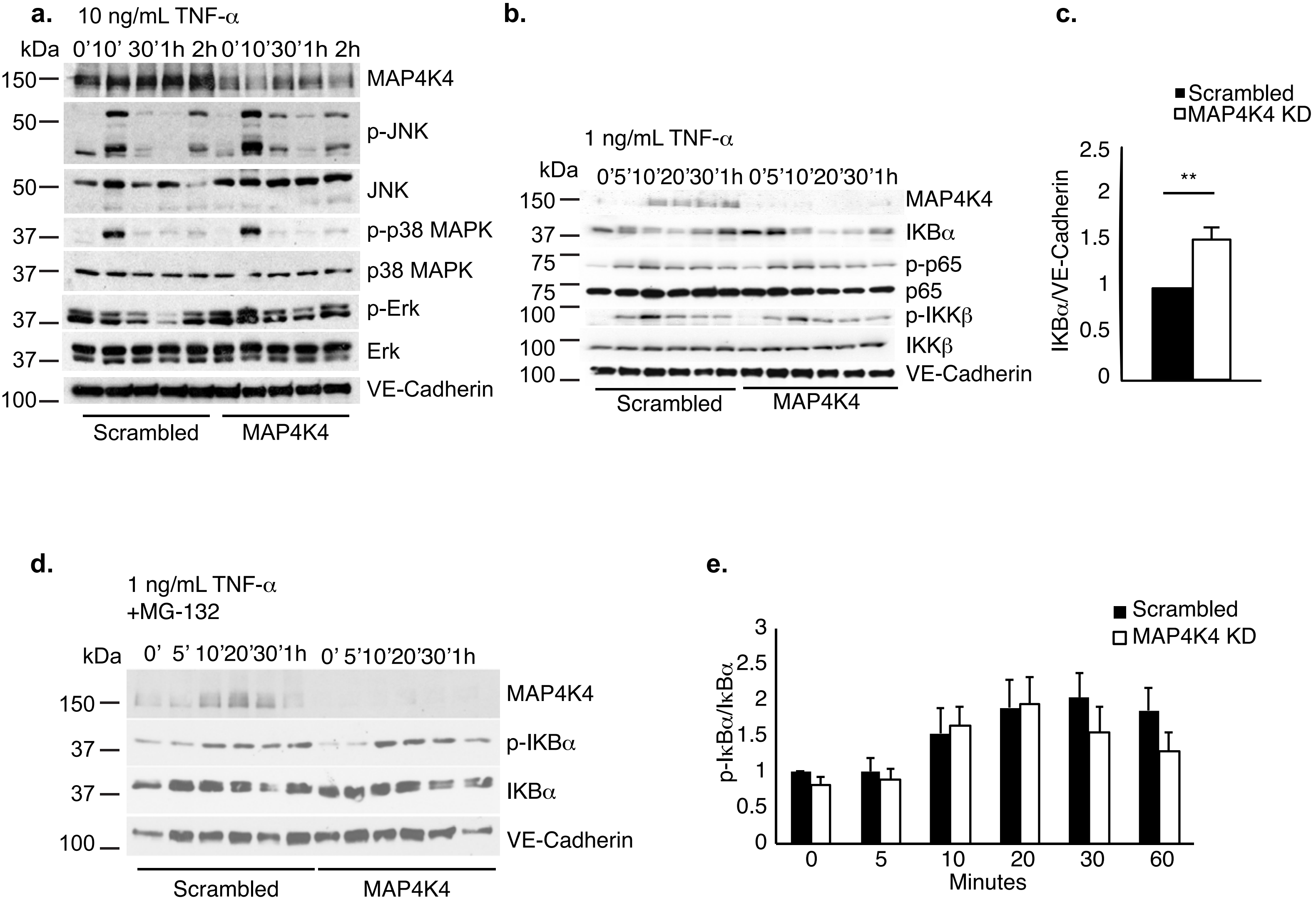

Supplementary Figure 2. EC MAP4K4 does not promote TNFα-induced MAPK or IKK activation. **a-e.** HUVECs were treated with scrambled or MAP4K4 siRNA and stimulated with 1 or 10 ng/mL TNF-α for the indicated times. **a.** Lysates were immunoblotted for MAP4K4, p-JNK, total JNK, p-p38 MAPK, total p38 MAPK, p-Erk, total Erk and VE-Cadherin. The data are representative of 4-5 independent experiments. **b.** Lysates were immunoblotted for MAP4K4, total IκBα, phospho-p65, total p65, phospho-IKKβ, total IKKβ, and VE-Cadherin. The data are representative of 4-10 independent experiments. **c.** Densitometric analyses represent the mean ± SEM of IκBα expression at time 0 as normalized to VE-Cadherin (**; $p<0.005$, N=4-10). **d.** HUVECs were pre-treated with MG132 prior to TNF-α stimulation. Lysates were immunoblotted for MAP4K4, phospho- IκBα, total IκBα, and VE-Cadherin. Data are representative of 5 experiments. **e.** Densitometric analyses represent the mean ± SEM of phospho-IκBα expression as normalized to total IκBα (N=5).

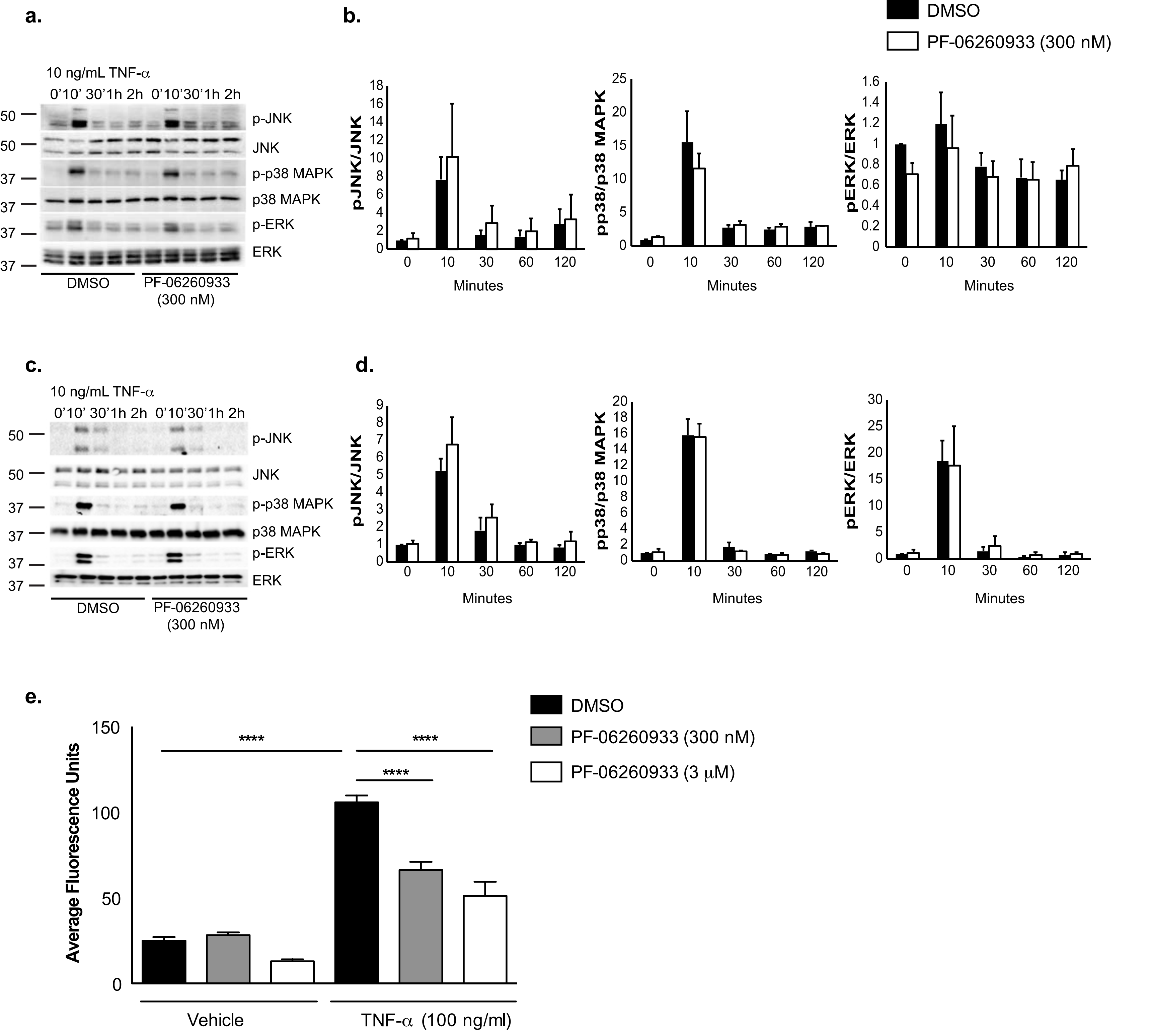

Supplementary Figure 3. MAP4K4 kinase inhibition does not affect MAPK signaling and ameliorates vascular permeability. a-b. HUVECs or **c-d.** Peritoneal macrophages derived from wild type mice were treated with DMSO or 300 nM PF-06260933 and stimulated with 10 ng/mL TNF-α for the indicated times. **a, c.** Lysates were immunoblotted for MAP4K4, p-JNK, total JNK, p-p38 MAPK, total p38 MAPK, p-Erk, and total Erk. The data are representative of at least 3 independent experiments. **b, d.** Densitometric analyses represent the mean ± SEM of (left) phospho-JNK expression as normalized to total JNK expression, (middle) phospho-p38 MAPK as normalized to p38 MAPK, and (right) phospho-Erk as normalized to total Erk (N=3). **e.** HAECs were pre-treated with DMSO or PF-06260933 as indicated, confluent cells were treated overnight with 100 ng/mL TNF-α or left untreated, and FITC labeled dextran that migrated through the HAEC monolayer was measured. The data represent the mean fluorescence intensity ± SEM (ANOVA ****; $p<0.0001$, N=3-4).

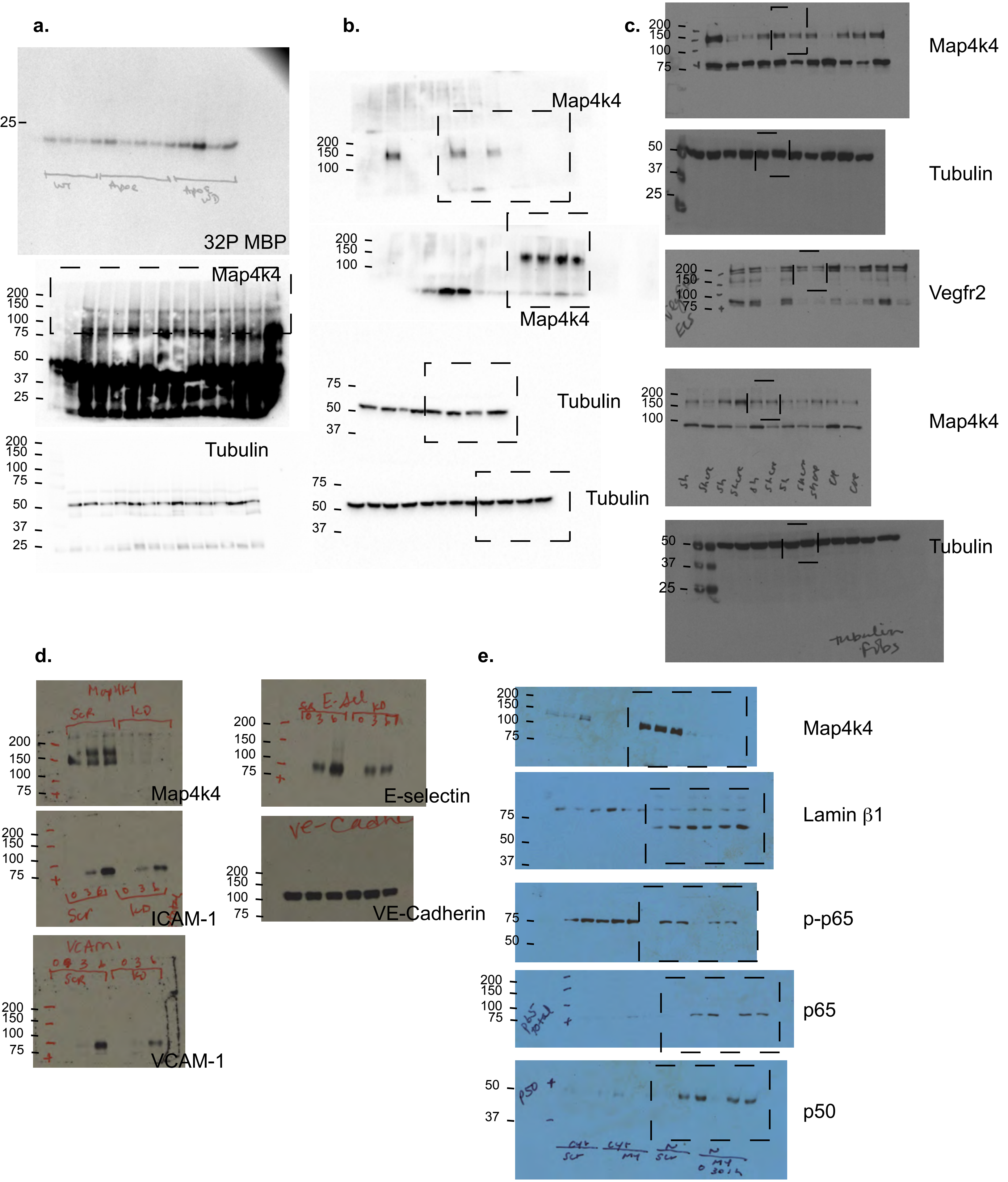

Supplementary Figure 4: Full gel scans for Figures 1-4. a. Gel scans for Fig. 1. **b.** Gel scans for Fig. 2. **c.** Gel scans for Fig. 3. **d.** Gel scans for Fig. 6g-h. **e.** Gel scans for Fig 6. i-j. Dashed boxes indicate lanes used if not entire gel.

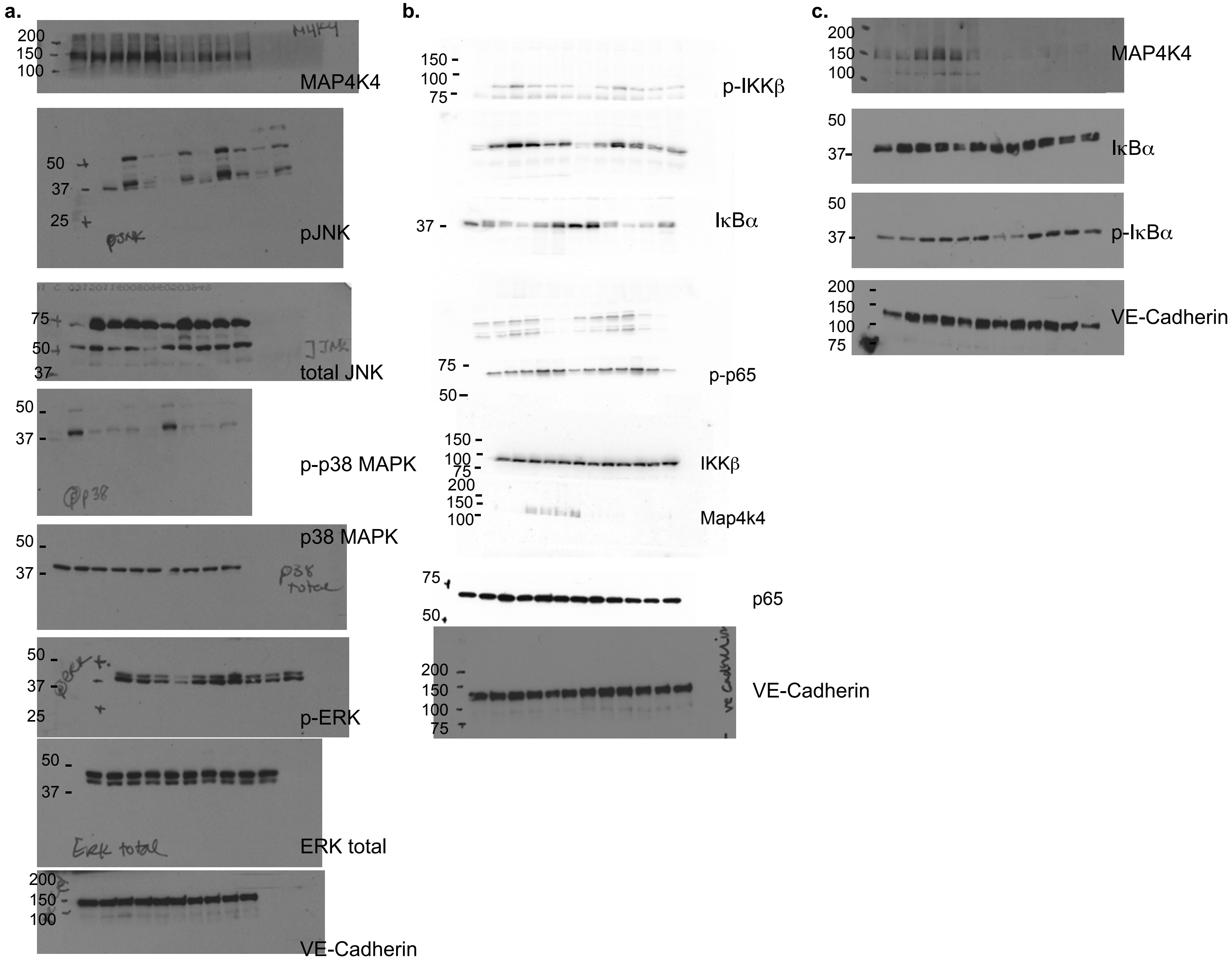

Supplementary Figure 5: Full gel scans for Supplementary Figure 2. a. Gel scans for Supplementary Fig. 2a. **b.** Gel scans for Supplementary Fig. 2b-c. **c.** Gel scans for Supplementary Fig. 2d-e.

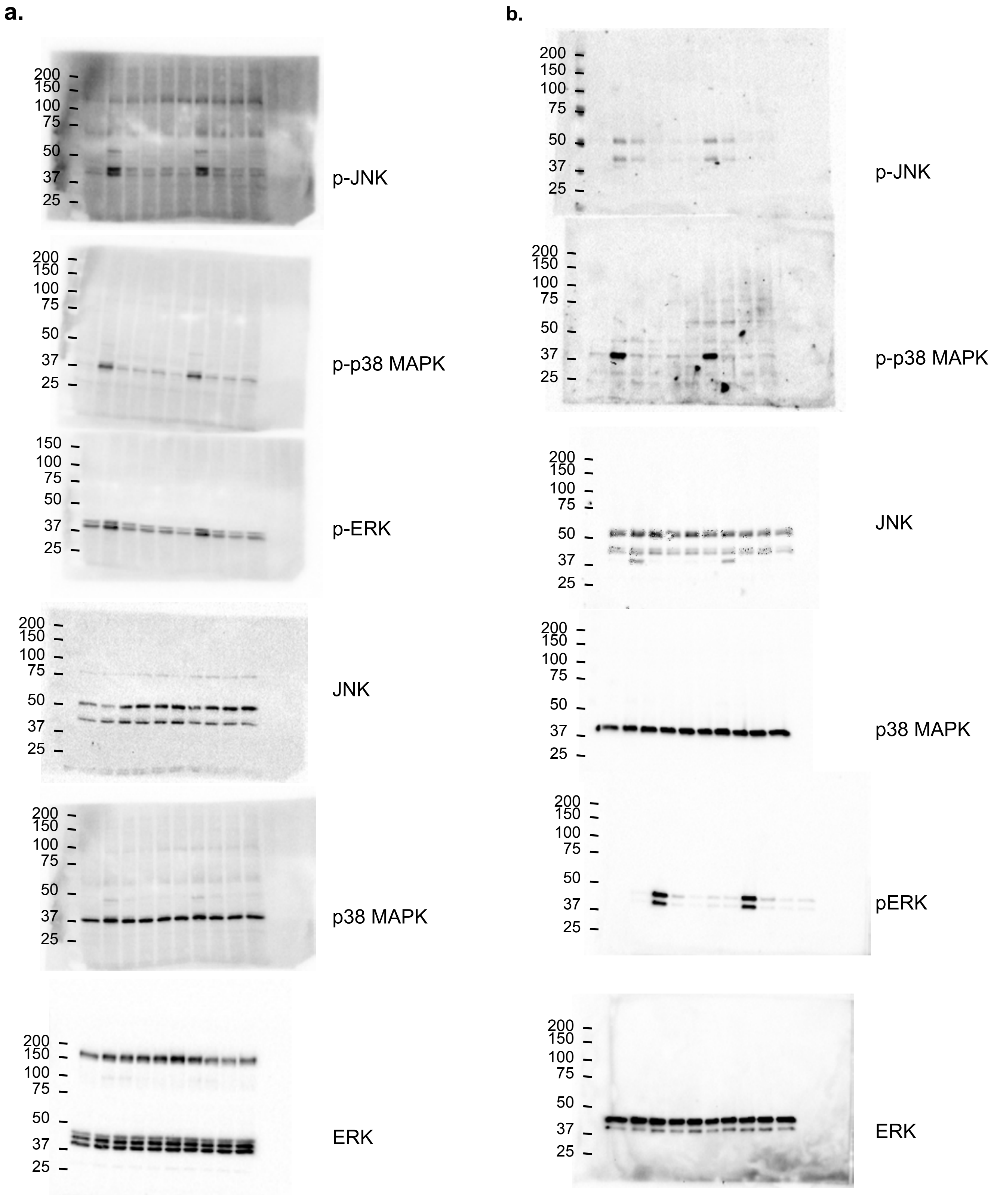

Supplementary Figure 6: Full gel scans for Supplementary Figure 3. a. Gel scans for Supplementary Fig. 3a-b. **b.** Gel scans for Supplementary Fig. 3c-d.

Supplementary Information

Supplementary Table 1

Plasma glucose and lipid levels in MAP4K4 KD or PF06260933-treated mice

	16 weeks Western diet		*Apoe*$^{-/-}$ prevention model		*Ldlr*$^{-/-}$ regression model	
	Control	MAP4K4 KD	Vehicle (water)	PF-06260933 (10 mg/kg)	Vehicle (water)	PF-06260933 (10 mg/kg)
Body weight (g)	29.0 ± 0.5	32.3 ± 0.9*	24.2 ± 0.5	24.9 ± 0.3	35.5 ± 1.1	33.8 ± 0.9
Glucose (mg/dL)	148.0 ± 26.3	146.4 ± 13.9	340.5 ± 21.7	266.3 ± 14.79 *	166.7 ± 6.0	139.0 ± 7.7**
Total TG (mg/dL)	135.9 ± 16.8	123.9 ± 12.7	159.5 ± 14.14	133.0 ± 13.69	264.5 ± 21.2	183.3 ± 13.6**
Total Cholesterol (mg/dL)	771 ± 67.5	803.0 ± 46.7	1529 ± 128.8	1424 ± 195.2	1613 ± 97.0	1236 ± 61.2**
HDL Cholesterol (mg/dL)	30.2 ± 5.2	35.6 ± 6.7	515.1 ± 35.1	502.7 ± 60.17	367.7 ± 15.9	387.2 ± 13.6
LDL Cholesterol (mg/dL)	245.4 ± 33.1	254.8 ± 20.9	1357 ± 117.5	1288 ± 181.1	1553 ± 96.5	1174 ± 64.0**

The data represent the mean ± S.E.M. (*; p<0.05, **; p<0.005, N=6-12**)**.

Supplementary Table 2

RT-PCR primer sequences

Gene	Forward	Reverse
**MAP4K4 (hum)**	GGGGAACGCTTCAGAGTGAG	GTGCGGTCAGATCAGCAGG
**ICAM-1 (hum)**	TCTGTGTCCCCCTCAAAAGTC	GGGGTCTCTATGCCCAACAA
**VCAM-1 (hum)**	ATGCCTGGGAAGATGGTCG	GACGGAGTCACCAATCTGAGC
**SELE (hum)**	GATGAGAGGTGCAGCAAGAAG	CTCACACTTGAGTCCACTGAAG
**RPLP0 (hum)**	CAGATTGGCTACCCAACTGTT	GGGAAGGTGTAATCCGTCTCC
**GAPDH (hum)**	ATGTTCGTCATGGGTGTGAA	GGTGCTAAGCAGTTGGTGGT
**Map4k4**	CATCTCCAGGGAAATCCTCAGG	TTCTGTAGTCGTAAGTGGCGTCTG
**Icam-1**	GTGATGCTCAGGTATCCATCCA	CACAGTTCTCAAAGCACAGCG
**Vcam-1**	AGTTGGGGATTCGGTTGTTCT	CCCCTCATTCCTTACCACCC
**Sele**	ATGAAGCCAGTGCATACTGTC	CGGTGAATGTTTCAGATTGGAGT
**Selp**	CATCTGGTTCAGTGCTTTGATCT	ACCCGTGAGTTATTCCATGAGT
**F4/80**	CCCCAGTGTCCTTACAGAGTG	GTGCCCAGAGTGGATGTCT
**Cd68**	CCATCCTTCACGATGACACCT	GGCAGGGTTATGAGTGACAGTT
**Ccl2**	TTAAAAACCTGGATCGGAACCA A	GCATTAGCTTCAGATTTACGGGT
**Cxcl1**	CTGGGATTCACCTCAAGAACAT C	CAGGGTCAAGGCAAGCCTC
**Ccl3**	TTCTCTGTACCATGACACTCTGC	CGTGGAATCTTCCGGCTGTAG
**Ccl4**	TTCCTGCTGTTTCTCTTACACCT	CTGTCTGCCTCTTTTGGTCAG
**Ccl5**	TCGAGTGACAAACACGACTGC	GCTGCTTTGCCTACCTCTCC
**Ccl7**	GCTGCTTTCAGCATCCAAGTG	CCAGGGACACCGACTACTG
**Cxcl9**	TCCTTTTGGGCATCATCTTCC	TTTGTAGTGGATCGTGCCTCG
**Cxcl10**	CCAAGTGCTGCCGTCATTTTC	GGCTCGCAGGGATGATTTCAA
**36b4**	TCCAGGCTTTGGGCATCA	CTTTATCAGCTGCACATCACTCAGA
**VCAM-1 (ChIP hum)**	TCAGCATTGTCCTTTATCTTTCC AG	ACTATTAACCCCTTCAGTTGCTCTC
**SELE (ChIP hum)**	CAAGAGACAGAGTTTCTGACAT CAT	TTTATAGGAGGGATTGCTTCCTGT G

Sequences are mouse unless specified.

Supplementary Table 3

Human clinical data

**Normal patient data**		
**Gender**	**Age**	**% Stenosis**
Male	84	N/A
Male/Female (pool of 4)	27-45	N/A
Male	44	N/A
**Atherosclerosis Patient data**		
**Gender**	**Age**	**% Stenosis**
Male	82	79
Female	65	99
Male	75	85
Male	63	99
Male	46	Unknown
